# Lexical Input in the Grammatical Expression of Stance: A Collexeme Analysis of the INTRODUCTORY *IT* PATTERN

**DOI:** 10.3389/fpsyg.2021.762000

**Published:** 2022-01-05

**Authors:** Zhong Wang, Weiwei Fan, Alex Chengyu Fang

**Affiliations:** ^1^School of Foreign Languages, Huazhong University of Science and Technology, Wuhan, China; ^2^Department of Linguistics and Translation, City University of Hong Kong, Kowloon, Hong Kong SAR, China

**Keywords:** the INTRODUCTORY *IT* PATTERN, collexeme analysis, *Epistemic* stance, *Deontic* stance, *Dynamic* stance, *Evaluation* stance

## Abstract

Previous research on the INTRODUCTORY *IT* PATTERN unveiled various lexical and grammatical aspects of its use as a grammatical stance device, including the range of the most frequently used adjectival and verbal stance lexemes, associated stance meanings, the most frequent sub-patterns, and the distinct uses in various contextual settings of the pattern. However, the stance meanings of the pattern, which are deeply rooted in the associated lexical resources, are still understudied. This study explores the meanings of the INTRODUCTORY *IT* PATTERN by referring to the stance meanings of the pattern associated with the adjectival and verbal lexemes that are statistically attracted to the pattern. The research samples were extracted from the British component of the International *Corpus* of English (ICE-GB). The samples were manually annotated for different stance types and a collexeme analysis was performed to identify the full range of stance lexemes statistically associated with the INTRODUCTORY *IT* PATTERN (collexemes). The results show that both adjectival and verbal collexemes are statistically and functionally significant for the delivery of discrete stance types/subtypes. Adjectival collexemes are frequently deployed for all four stance types: *Epistemic* stance, *Evaluation* stance, *Dynamic* stance, and *Deontic* stance, while verbal collexemes are valuable lexical resources for the *Epistemic* stance, as their use entails modalized evidentiality, pointing to epistemic judgment of the writer-speaker toward events/propositions. Close examination of the use of adjectival and verbal collexemes identified three fundamental meanings of the INTRODUCTORY *IT* PATTERN. First, the pattern is inherently evaluative as it tends to attract more lexemes with evaluative meanings and associates evaluative meanings with superficially non-evaluative lexemes. Second, it features a scalarized expression of diversified stance types/subtypes, thus, especially reflective of the scalarized semantic feature of stance expression. Third, it connotates an overwhelmingly positive likelihood judgment. The article concludes by discussing the limitations of this study.

## Introduction

The expression of writer-speaker stance is crucial for language use (Biber et al., 1999, p. 966; Du Bois, 2007, p. 139). Linguistic devices available for stance expression include evaluative *lexis* (Biber et al., 1999, p. 966; Biber, 2006), grammatical constructions, and meta-discourse features operating at the interactive or interpersonal level (Hyland, 2004, 2017). Most recently, there has been an increasing interest in *corpus*-based approaches to evaluative *lexis*, e.g., Pérez Blanco (2020) and Orr and Ariel (2021), which are focused on stance-marking adjectives such as *clear*, *obvious*, *evident*, and *true*. Yet, as observed in Biber (2006), a research challenge lies in the fact that these stance markers are not always evaluative. Hunston (2007) quotes the example of *scholarly* and points out that its meaning in *this is a fine, scholarly work* is different from that in *a scholarly study*. It is evident that even very large *corpora* cannot exhaustively itemize all the evaluative *lexis* of a given stance type. In comparison, grammatical stance devices, which represent clear cases of stance expression (Biber et al., 1999; Biber, 2006), offer a promising alternative in the study of a writer-speaker stance. They are particularly suitable for the quantitative search of the full range of phraseological expressions for various stance types/subtypes based on *corpus* data (Groom, 2005; Biber, 2006; Hunston, 2007, p. 35).

Among the various grammatical devices, in particular, the INTRODUCTORY *IT* PATTERN features a distinctive sentence structure with two subjects: an anticipatory *it* as the provisional subject and a post-verbal clause (including *that*-clauses, *to*-infinitive clauses, -*ing* clauses, and *wh*-clauses) as the notional or semantic subject (cf. Quirk et al., 1985, p. 1391). The pattern involves “two distinct linguistic components, one presenting the stance and the other presenting a proposition that is framed by that stance” (Biber et al., 1999, p. 970), which are actualized by its main clause and the clausal notional/semantic subject, respectively. Thus, the INTRODUCTORY *IT* PATTERN is also acknowledged as a grammatically unified convention that integrates the writer-speaker stance and the assessed propositions/events (Couper-Kuhlen and Thompson, 2008). Research on this pattern also argues that the expression of stance *via* the finite main clause of the INTRODUCTORY *IT* PATTERN allows for flexibility in the deployment of mood, voice, modal verbs, and various adverbials (Herriman, 2000a,b), which, in turn, add to the informativeness of the INTRODUCTORY *IT* PATTERN (Herriman, 2000a,b).

The INTRODUCTORY *IT* PATTERN has been widely investigated for its stance-marking function. The most widely investigated issue is the form-meaning correlation between the types of stance meanings expressed by the main clause and the syntactic categories of the notional subject clause (e.g., Mair, 1990; Collins, 1994; Herriman, 2000a; Zhang, 2015). For example, research has revealed that the expression of *Dynamic* stance, including the Ease-of-performance and Circumstance stance subtypes, is associated with infinitival notional subject clauses, while the *Epistemic* stance correlates with *that*-clause (Herriman, 2000b). Alternatively, researchers also analyze its sub-patterns like the *it verb-link* adjective (ADJ) *to*-infinitival pattern and *it verb-link* ADJ *that*-clause pattern in genre-specific and discipline-specific academic writings (Biber et al., 1999, pp. 675, 721; Charles, 2000; Groom, 2005; Roomer, 2009). Research findings suggest that, for example, genre/register influences the distribution and the dominant meanings of the two sub-patterns. Thus, research articles and book reviews in the fields of history and literature demonstrate a discrete preference for phraseology in the ADJ slot and a different preference for the two sub-patterns (Groom, 2005).

More recently, collexeme analysis (Stefanowitsch and Gries, 2003) has been applied to the INTRODUCTORY *IT* PATTERN (Hilpert, 2014) to reveal its semantic connotations. Hilpert (2014) performed a collexeme analysis on the *it’s* ADJ *to*-infinitive sub-pattern of the INTRODUCTORY *IT* PATTERN. The study serves as a case study demonstrating the operational process of the collexeme analysis, by which the full range of lexemes that are significantly attracted (collexemes) to the ADJ slot in the sub-pattern was identified. The decontextualized lexical meanings of the 20 most attracted collexemes are found to form four meaning groups: ease, possibility, advisability, and importance. However, the finding abstracts away from the linguistic context, resulting in an inaccurate categorization of meaning groups. For example, *possible* and *impossible* were spotted as strongly attracted collexemes and were classified into the “possibility” group by Hilpert (2014). However, when occurring in the ADJ slot of the *it be* ADJ *to*-infinitival sub-pattern, they assess unrealized events encoded by to-infinitival clauses (Mair, 1990; Herriman, 2000a). Along this line, they could only assess unrealized events as potential/easy/difficult to actualize. Accordingly, *possible* and *impossible* belong to the “ease” group rather than the “possible” group. This conflation of different stance meanings could be sufficiently reduced if the grouping of collexemes is supported with contextualized semantic analysis. Nonetheless, the ideology behind the collexeme analysis is in line with the *Semantic Coherence Principle* in Construction Grammar (Goldberg, 1995, p. 50) in maintaining that the meanings of collexemes should be semantically coherent with the pattern that attracts them. Thus, the collexeme analysis, adequately supported with semantic analysis of collexemes, has the prospect of revealing the meanings of constructions and patterns.

Compared to previously mentioned methods, collexeme analysis assumes that the lexical items, which are significantly associated with the pattern, indicate its semantic connotations. In contrast, other methods presume that the lexical items with the highest observed frequency represent the prototypical meaning expressed by the pattern or other constructions. The significant association is based on relative frequency, while the observed frequency adopted by other methods is absolute frequency. Between significant association and high-frequency counts, the former is based on the cognitive entrenchment of language users toward the meanings of the pattern (Gilquin, 2013). The latter, however, could result from an even distribution of high-frequency lexical items that are not correlated to the pattern. As a result, collexeme analysis has greater statistical rigor and can be a powerful semantic indicator of how language users choose/fill in lexemes for the INTRODUCTORY *IT* PATTERN in actual language use (Gries et al., 2005; Stefanowitsch, 2006).

As suggested by Hilpert (2014), the semantic parsing of collexemes should be performed in the process of collexeme analysis. The meanings of collexemes should be “examined within their linguistic contexts rather than just by looking at a list of these collexemes” (Hilpert, 2014, p. 397). A similar concern is echoed by Gilquin (2013) and Deshors (2017). However, due to different research interests and, often with limited resources, researchers rarely perform a full-scale semantic analysis on collexemes (Hilpert, 2014; Larsson and Kaatari, 2019). Van Linden (2010), for instance, focused exclusively on the *Deontic* stance expressed *via* the INTRODUCTORY *IT* PATTERN. Even so, there is the common understanding that extensive semantic annotation of collexemes will shed light on how language users conceptualize the meanings of the INTRODUCTORY *IT* PATTERN. Thus, helping to explain its use as a stance-marking device.

This study will perform a collexeme analysis of the INTRODUCTORY *IT* PATTERN to explore its stance meanings. Specifically, lexemes that are statistically attracted to the predicate position of the pattern will be semantically analyzed. To support the analysis, the stance meanings expressed by the predicates of the INTRODUCTORY *IT* PATTERN will be manually classified to guarantee a proper semantic interpretation of the contextualized stance meanings. Discussions will focus on how the stance meanings are expressed based on the actual use of collexemes within the INTRODUCTORY *IT* PATTERN, leading to generalizable conclusions of its semantic connotations.

## Stance Marking by the Introductory *IT* Pattern

Stance is the attitude, feelings, judgments, or commitment of writer-speakers (Biber and Finegan, 1989) toward entities and propositions. It is one of the essential communicative purposes of the use of the language (Biber et al., 1999). The major components of stance include attitudinal stance/affect, *Epistemic* stance, and dialogic/interactional stance (Biber et al., 1999; Biber, 2006; Wharton, 2012). *Corpus* approaches to stance focus on lexical and grammatical patterns in academic discourse (Charles, 2003; Hyland and Tse, 2005; Biber, 2006; Chan, 2016; Pérez-Paredes and Bueno-Alastuey, 2019; Vasiliki et al., 2020), academic discourse across languages (Warchał, 2015), and legal genres under different legislative systems (Cheng and Cheng, 2014; Koźbiał, 2020). Under such interests, the range of stance meanings expressed by various stance devices has been discussed. Among all grammatical devices of stance, adverbials and complement clause structures mark the clearest cases of stance expression directed toward the events/propositions (Biber et al., 1999, pp. 972 – 975; Biber, 2006).

Following the line of complement clause structures, the INTRODUCTORY *IT* PATTERN, as a member of such structures (Biber et al., 1999, p. 969), has received a great attention from researchers. Within this pattern, as in sentence (1), the underlined *that* clause is the extraposed subject clause, as well as the semantic subject. Meanwhile, the predicate *seems*, meaning *seems true/likely/to be the case* (Dixon, 2005), is a writer-speaker comment on the content of the extraposed subject clause (Biber et al., 1999, p. 970; Herriman, 2000a,b; Hamawand, 2007).

(1) Indeed, it seems that girls very quickly replaced boys at this task (Biber et al., 1999, p. 973).

The INTRODUCTORY *IT* PATTERN features impersonal stance expression (Charles, 2000, 2006; Herriman, 2000a; Biber, 2006), resulting from its impersonal grammatical subject *it* and the usually implicit attribution to the source of the stance (Charles, 2000, 2006). Besides, it displays three unique features. First, it is important in academic writings (Biber et al., 1999; Hewings and Hewings, 2002; Larsson, 2016, 2017) as it hedges authorial stance (Rowley-Jolivet and Carter-Thomas, 2005), and demonstrates the formality of academic writing (Larsson and Kaatari, 2019). Second, the stance expressed at the construction-initial position is the thematic starting point that frames the interpretation of a reader-hearer of the following extraposed subject clause (Collins, 1994; Gómez-González, 1997, 2001). Third, the expressed stance presented as the theme that delivers unmarked old information (Halliday, 1967; Halliday and Matthiessen, 2004) cannot be conveniently disputed by the reader-hearers (Hoey, 2000). These features make the INTRODUCTORY *IT* PATTERN an important stance device.

Researchers have proposed different classifications of stance meanings expressed by the INTRODUCTORY *IT* PATTERN (Gómez-González, 1997, 2001; Herriman, 2000a; Kaltenböck, 2005; Langacker, 2009) or by some of its sub-patterns (Mair, 1990; Collins, 1994; Groom, 2005; Roomer, 2009; Hilpert, 2014). Among the various classifications, Herriman (2000a) offers a systematic and inclusive framework that covers almost all stance types proposed by other researchers. According to Herriman (2000a), stance expressed by the INTRODUCTORY *IT* PATTERN can be classified into four types: *Epistemic* stance, *Deontic* stance, *Dynamic* stance, and *Evaluation* stance. These four types respectively correspond to the likelihood judgment, the desire/need to realize events/propositions, the natural laws or empirical circumstances to be obeyed, and various value judgments. Each type can be divided into various subtypes: *Epistemic* stance is divided into Truth, Existence, and Perception; *Deontic* stance into Volition and Obligation; *Dynamic* stance into Circumstance, Ease-of-Performance/Potentiality, and Human Attribute; *Evaluation* stance into General Evaluation, Appropriateness, Emotive Reaction, Significance, Responsibility, and Frequency. Among these subtypes, Human Attribute assesses the personal traits of the subject in the extraposed subject clause. It is only scarcely identified in the language samples of Herriman (2000a), as is the case in our research samples from the British component of the International *Corpus* of English (ICE-GB). Therefore, the stance subtype of Human Attribute is omitted in this analysis.

Although Herriman aims at a comprehensive stance framework, her framework is considered solely applicable to adjectival and nominal predicates of the INTRODUCTORY *IT* PATTERN (Zhang, 2015). Verbal predicates are treated with a different framework (e.g., Francis et al., 1996; Biber et al., 1999; Biber, 2006) in empirical investigations (Charles, 2006; Zhang, 2015). The inadequacy lies in Herriman’s inconsistent analysis of verbal predicates which express evidentiality, defined as the indication about the source of information. The framework of Herriman (2000a) addresses two types of verbal predicates as *Epistemic* expressions, which comment on (1) the existence/coming into existence, and (2) the perception of events/propositions. The two meanings indicate evidentiality because they point the sources of information to the visual observation and the sensory perception, respectively, of writer-speaker. However, Herriman (2000a) does not include the verbs of reporting/communication, another evidentiality expression addressed in Biber et al. (1999) and Zhang (2015), which are frequently attested in the main clause of the INTRODUCTORY *IT* PATTERN (Francis et al., 1996; Biber et al., 1999; Charles, 2006).

The inadequate treatment of evidentiality takes us to two further issues, one about the different sub-categories of evidentiality, the other about the boundary between evidentiality and *Epistemic* stance. The verbal phrases describing “the *Existence* or coming into existence” of events/propositions (Herriman, 2000a, p. 585) are expressions of Direct evidentiality defined as an indication that events/propositions are the direct observation or perception of writer-speakers (Plungian, 2001, 2010). The same applies to verbal phrases of perception, if they remain at the level of sensory perception (Plungian, 2001, 2010) without extending to the interpretation of some other events/propositions (Field, 1997; Plungian, 2010; Whitt, 2010, 2011). In the former case, verbs of perception express Direct evidentiality while in the latter case, they express Indirect evidentiality, which is an indication about the indirect access of writer-speaker to information (Plungian, 2001, 2010). Besides, verbs of reporting/speaking express Reported evidentiality (Plungian, 2001, 2010) that attributes the source of information to someone else. Therefore, the main clause predicates of the INTRODUCTORY *IT* PATTERN can express the three commonly acknowledged sub-categories of evidentiality: Direct, Indirect, and Reported evidentiality (Plungian, 2001, 2010).

Among the three sub-categories, Indirect evidentiality is the domain where evidentiality and *Epistemic* stance intersects (Plungian, 2010; Boye, 2012; Pérez Blanco, 2020; Marín-Arrese, 2021). This intersection is grounded on the general conception that indirectly obtained evidence is less reliable than those directly observed. In other words, Indirect evidentiality indicates that writer-speakers infer from other evidence or make deductions (e.g., *it looks as if; it might be that*) to support the current events/propositions (Plungian, 2001, 2010). This way of presenting events/propositions can be deemed as an expression of “epistemic necessity” that evaluate the events/propositions as probable (Plungian, 2010), an expression of the epistemic justification of writer-speakers (Boye, 2012), or epistemic attitude (Marín-Arrese, 2021).

The evaluative evidentiality is also addressed as “modalized” evidentiality (Plungian, 2001), where writer-speakers indicate the source of information and express their probability judgment. Along this line of modalized evidentiality, verbs of perception can mark indirect evidentiality, as their meanings can extend to the perception of the non-physical/internal world of reasoning or inference (Whitt, 2010, 2011) rather than a direct observation of the physical world. In addition, reporting verbs can mark the modalized evidentiality as well when they are pre-modified by modal verbs (Plungian, 2010). Interestingly, despite the expression of Direct evidentiality, verbal predicates of the INTRODUCTORY *IT* PATTERN, with the meaning of existence/occurrence, can assess events/propositions as “logically the case” (Francis et al., 1996, p. 519), thus, delivering *Epistemic* stance.

Putting all pieces together, the inconsistency problem of the framework of Herriman (2000a) can be addressed *via* an investigation of modalized evidentiality. Those modalized evidential meanings should be accommodated into the stance framework while those non-modalized evidential meanings should be excluded. Apart from verbal predicates that indicate evidentiality, the framework can plausibly situate other verbal phrases into appropriate stance types/subtypes. For example, *matter*, *help*, and *surprise* are suitably categorized as verbs of Significance, General Evaluation, and Emotive Reaction, respectively. Therefore, the resolution of the inconsistency problem is important to piece together an adequate description of the stance meanings of the INTRODUCTORY *IT* PATTERN, which is precisely within the scope of this study.

Guided by previous research findings, we focus our attention on adjectival and verbal predicates as other predicates, including nominal and prepositional predicates, are rarely attested in the INTRODUCTORY *IT* PATTERN (Mair, 1990; Francis et al., 1996; Kaltenböck, 2000). Between the two types of selected predicates, the adjectival predicates are acknowledged to be the prevalent type (Kaltenböck, 2000; Groom, 2005; Hilpert, 2014). Meanwhile, the verbal predicates have also received considerable attention (Francis et al., 1996; Charles, 2000, 2006; Dixon, 2005; Zhang, 2015). An investigation considering both the adjectival and verbal predicates helps depict a complete picture of the stance semantics of the INTRODUCTORY *IT* PATTERN. The following research questions will guide the rest of this research.

1. How are the adjectival and verbal collexemes used to express stance in the INTRODUCTORY *IT* PATTERN?
2. What are the stance meanings of the INTRODUCTORY *IT* PATTERN as indicated using collexemes?

## Research Data and Methods

### Research Samples

Samples for this research were extracted from the International *Corpus* of English-Great Britain (ICE-GB; Greenbaum, 1996). Sub-component; a one-million-token *corpus* comprised of written and transcribed spoken discourse from native British-English speakers. The corpus contains 300 spoken and 200 written text units. Among the 300 transcribed spoken texts, 50 scripted speech texts are more characteristic of a written discourse and, thus, can be classified into written discourse (Kaltenböck, 2005). In this sense, the ICE-GB consists of an equal share of spoken and written texts.

The ICE-GB is intensively marked with grammatical features. The anticipatory *it* of the INTRODUCTORY *IT* PATTERN is marked with “PRSU” and “ANTIT” (“provisional subject” and “anticipatory *it*,” respectively), and the extraposed subject clause is marked with “NOSU, CL” (for “notional subject clause”). Meanwhile, the main clause predicates are also syntactically parsed. These markups are helpful for the syntactic parsing of the INTRODUCTORY *IT* PATTERN. By locating the “NOSU, CL” markup in each text file in ICE-GB, we identified all the INTRODUCTORY *IT* PATTERNs with Python 3.6.5. In addition, guided by the text unit or sentence boundary markup in the ICE-GB, we extracted the preceding seven sentences totaling to over 100 tokens as the pre-context, which is the basis for a proper interpretation of stance expression (Du Bois, 2007, p. 149).

After manual selection, we ended up with 1,746 cases, among which, 12 cases had coordinated predicate groups. Each coordinated predicate group (e.g., *it may be too late or not desirable to make any change*) was treated as two predicates (e.g., *may be too late* and *may be not desirable*). Therefore, the total number of predicates was 1,758. Among them, 1,042 (59.27%) were adjectival predicates and 472 were verbal predicates (26.85%), thus, constituting 86.12% of all main clause predicates. The others were nominal predicates (10.07%) and adverbial predicates (3.8%). As this study focuses on adjectival and verbal predicates to overcome data sparsity, we, therefore, concentrate on the combined total of 1,514 adjectival and verbal predicates.

### Collexeme Analysis

Collexeme analysis analyzes the attractions between lexemes and various abstract (e.g., passivation, dative) or specific constructions by calculating collostruction strength, which is “the degree to which particular slots in a grammatical structure prefer, or are restricted to, a particular set or semantic class of lexical items” (Stefanowitsch and Gries, 2003, p. 211). The analysis has been performed with the R script from Gries (2007). Methods generally used to calculate collostructional strength (CLS) in the R script include the Fisher-Yates Exact test, log-likelihood, chi-square, and odds ratio (Gries, 2007). Following Larsson and Kaatari (2019), this study applied the Fisher-Yates Exact test to statistically measure the CLSs between lexemes and grammatical patterns. Regardless of the choice of the statistical method, lexemes whose CLSs are above the threshold value are considered significantly attracted to the target slot and are called “collexemes” (Stefanowitsch and Gries, 2003).

### Research Procedures

First, two native English speakers were recruited as the annotators for the manual classification. The annotation tool was the UAM CorpusTool 3.3s. The classification was based on the interpretative result by annotators of what the main clause predicate commented about the content of the extraposed subject clause. The scope of the main clause predicate is exemplified by the underlined segment in (2). For all example sentences starting from (2), the source of the example sentence is provided at its end in the brackets (e.g., “s2b-032#039”) to indicate the name of the *corpus* file (e.g., “s2b-032#039”) and the exact index of the sentence (e.g., “039”).

To obtain reliable annotation results, the researchers trained the two annotators for the manual classification. The two annotators were first imparted necessary grammatical knowledge about the INTRODUCTORY *IT* PATTERN, and were then taught about the framework of Herriman (2000a) and the operational procedures of the annotation tool. For the few rounds of training, the annotators had several hands-on annotations of the random samples and their inter-annotator agreements (IAA) were calculated with Krippendorff’s alpha. After the IAAs were steadily above 0.8, which is the cut-off point for good agreements (Krippendorff, 2019), the training was considered successful. The two annotators were then given all the samples of the INTRODUCTORY *IT* PATTERN for annotation.

(2) (a) It is interesting to discover that it is not a very great attraction (s2b-032#039).
   (b) It is fairly common to use symbolic representations (s2a-060#091).
   (c) It is quite possible that the next time it is used, the results will be different (w2d-017#013).
   (d) It cannot be right that these actions should go any further (w2e-007#091).

After the annotation was completed, the results were statistically analyzed to ensure their validity. The raw agreement for the annotation was 0.904 and the IAA was 0.884 (>0.8), suggesting good and reliable results. The disputed cases (*n* = 151) were then discussed by the two annotators to reach agreements. Six cases could not be agreed upon due to the different interpretations of the two annotators of the same cases. These cases were then arbitrated by the researchers.

Second, a collexeme analysis was performed on the INTRODUCTORY *IT* PATTERN. One vital step was the selection of the target slot in the main clause predicate of the pattern. Guided by our research goal of revealing the stance meanings of the INTRODUCTORY *IT* PATTERN, the target slot should host the essential stance meaning of the main clause predicate. For our target construction, the two subtypes of INTRODUCTORY *IT* PATTERN with adjectival and verbal predicates, we selected the predicate head position as the target slot. In this regard, the two subtypes of the pattern can be expressed as *it* verb-link X_ADJ_ and *it* X_VERB_, within which the X_ADJ_/X_VERB_ is the predicate head and the verb-link is the linking verb, such as copular verbs. In addition, other linguistic elements in the main clause predicate might include modal auxiliaries, semi-modals, adverbs, or negation devices. Thus, within the example sentences in (2), the predicate heads are *interesting*, *common, possible*, and *right*. In these and other cases, X_ADJ_ represents the essential semantic content of the main clause (Mair, 1990; Larsson, 2016).

However, the X_VERB_ is slightly different. It can be in an active or passive voice and can be an uncomplemented verb, a verb with various forms of complementation, or an idiomatic verb phrase (e.g., *turn out*, *make sense*). Accordingly, the X_VERB_ alone might represent the essential semantic content of the verbal predicate like the underlined verbs in *it follows that* and *it was found that*. In this case, the X_VERB_ could be modified by auxiliaries or adverbials, or it could be negated, but the meaning expressed by the whole predicate is closely associated with the meaning of X_VERB_ (Goldberg, 2006, p. 28). Alternatively, the X_VERB_ can be verbs with general meanings (Dixon, 2005; Goldberg, 2006), or can bear less semantic weight than their complements, like the *come* in *it has come to my attention*. Such cases are rare (*n* = 4). Meanwhile, the range of complementation to the general verbs is restricted. Thus, when used as a collexeme of the INTRODUCTORY *IT* PATTERN, *come* only occurs in the phrasal form of *come to my attention/notice* (*n* = 3), meaning *being perceived*. The restricted use of *come* is associated with the grammatical role of the *come* phrase as the predicate of the INTRODUCTORY *IT* PATTERN because the clausal semantic subject of the phrase is obviously incapable of *coming* (Dixon, 2005), but of *being perceived*.

Likewise, for other verbs that do not represent the complete essential meanings of the predicates, their occurrence in the X_VERB_ slot is strongly associated with one corresponding stance meaning. For example, the verb *take* in X_VERB_ signals the expression of Circumstance stance subtype, as it is almost invariantly used to comment on empirical conditions (e.g., *it takes us years*). Other examples include *annoy* for Emotive Reaction (e.g., *it annoys me to*), and *dawn* for Perception (e.g., *it dawned upon her that*). Thus, each verb occurring in the X_VERB_ slot is closely associated with one subtype of stance (Goldberg, 2006, p. 28). Therefore, it is fair to say that in the case of the INTRODUCTORY *IT* PATTERN, the X_VERB_ can represent the meaning of the main clause predicate.

Therefore, the predicate head slot (X_ADJ_ and X_VERB_) of adjectival and verbal predicates can represent the essential semantic content of the two predicates and, thus is an ideal target slot for collexeme analysis.

After the target slot was settled, the main clause predicate heads were manually selected and were lemmatized into lexemes. The linguistic data required for a collexeme analysis include the observed frequency count of each lexeme in the target slot, the *corpus* frequency count of each lexeme, the observed frequency count of the INTRODUCTORY *IT* PATTERN, as well as the *corpus* size (Stefanowitsch and Gries, 2003; Gries, 2007; Hilpert, 2014). Based on these data, the collexeme analysis was then performed with an R script (Gries, 2007) to calculate the CLSs of lexemes that occur in the target slot of the INTRODUCTORY *IT* PATTERN. The significant CLS threshold was set to >3 (*p* < 0.001) to ensure a high linguistic significance of collexemes, and, therefore, to render lexemes with CLS values that are higher than 3, as the valid collexemes, for our analysis. Consequently, we ended up with 124 collexemes that accounted for 82.17% of the stance expression by the INTRODUCTORY *IT* PATTERN, with adjectival and verbal predicates.

Apart from CLS values, the dispersion information of collexemes was also considered to rule out misguided findings due to the uneven distribution of the INTRODUCTORY *IT* PATTERN (Gries, 2008, 2019). The measurement of dispersion was *1-DP* (Gries, 2008) and its values for all the 124 collexemes were around 0.8, suggesting an even distribution of the INTRODUCTORY *IT* PATTERN in ICE-GB.

Finally, the 124 collexemes were checked for their association with different stance subtypes according to our annotation results. As the results suggested, out of the 124 collexemes, 109 featured a strict one-to-one association with the stance subtypes. For example, *interesting* is solely used to denote an Emotive Reaction [see (2a)], *common* for Frequency [see (2b)], and *right* for Appropriateness [see (2d)]. Seven other collexemes are primarily used to express one stance subtype but can be used to express other subtypes as well. The two groups of collexemes, totaling to 116 collexemes, will be reported with their associated stance types/subtypes in the following two sections.

The remaining eight collexemes are not prone to be associated with specific stance subtypes. Instead, they are indiscriminately used to express multiple subtypes of a stance with no statistical inclinations. These collexemes occurred 13 times in the target slot. Their multiple uses resulted from their polysemous nature and the differing linguistic circumstance. For example, *extraordinary* could mean *exceedingly good* or *strange* in different linguistic contexts, thus, denoting General Evaluation and Appropriateness, respectively. However, a small number of such cases will be insufficient to reveal about the stance meaning of the INTRODUCTORY *IT* PATTERN. Thus, these collexemes will not be discussed in the following sections.

## The Adjectival and Verbal Collexemes

Table 1 reports the use of adjectival and verbal collexemes for the expression of stance *via* the INTRODUCTORY *IT* PATTERN. In Table 1, the percentages that are marked in **bold** display are the prevalent association with adjectival collexemes, while the underlined percentages indicate a primary association with verbal collexemes. As can be seen from Table 1, Existence, Perception, and Volition were all expressed by verbal collexemes (100%), along with most Circumstance (77.08%) stance subtypes. In contrast, Obligation, Ease-of-Performance, and Frequency were solely denoted by adjectival collexemes. Meanwhile, one subtype of *Epistemic* stance, Truth, was mostly expressed by adjectival collexemes (67.13%), along with almost all the *Evaluation* subtypes, excluding Frequency, constituting 86.36 to 95.96% occurrences of these subtypes.

**TABLE 1:** Adjectival and verbal collexemes for stance expression.

Stance Types	Stance subtypes	Adjectival Collexemes			Verbal Collexemes			Total	
		Types	Counts	Percent	Types	Counts	Percent	Types	Counts
*Epistemic* Stance	Existence	0	0	0.00%	5	25	100.00%	5	25
	Perception	0	0	0.00%	7	123	100.00%	7	123
	Truth	17	194	**67.13%**	16	95	32.87%	33	289
	*Total*	*17*	*194*	*44.39%*	*28*	*243*	*55.61%*	*45*	*437*
*Deontic* Stance	Volition	0	0	0.00%	2	7	100.00%	2	7
	Obligation	3	57	**100.00%**	0	0	0.00%	3	57
	*Total*	*3*	*57*	*89.06%*	*2*	*7*	*10.94%*	*5*	*64*
*Dynamic* Stance	Circumstance	2	11	22.92%	2	37	77.08%	4	48
	Ease-of-Performance	5	245	**100.00%**	0	0	0.00%	5	245
	*Total*	*7*	*256*	*87.37%*	*2*	*37*	*12.63%*	*9*	*293*
*Evaluation* Stance	Appropriateness	23	95	**95.96%**	1	4	4.04%	24	99
	Emotive Reaction	9	51	**94.44%**	1	3	5.56%	10	54
	Frequency	4	15	**100.00%**	0	0	0.00%	4	15
	General Evaluation	11	125	**94.70%**	1	7	5.30%	12	132
	Significance	5	76	**86.36%**	2	12	13.64%	7	88
	*Total*	*52*	*362*	*93.30%*	*5*	*26*	*6.70%*	*57*	*388*
*Total*		*79*	*869*	*73.52%*	*37*	*313*	*26.48%*	*116*	*1182*

*Note: In the “Percent” column of “Adjectival Collexemes,” the percentages in bold show a prevalent use of adjectival collexemes for the expression of different “Stance Subtypes”. Likewise, in the “Percent” column of “Verbal Collexemes,” the underlined percentages demonstrate the overwhelming use of verbal collexemes for the expression of “Stance subtypes”.*

In addition, Table 1 also shows that 28 out of the 37 total verbal collexemes were used as *Epistemic* collexemes, accounting for 243 out of the 313 occurrences (77.64%) of verbal collexemes. Conversely, a large portion of the *Epistemic* stance and its subtypes were expressed by verbal collexemes. The portion has reached 100% for the subtypes of Existence and Perception, and 32.87% for the subtype of Truth. As for adjectival collexemes, they were widely used to denote all the four stance types. In particular, they were most prominently used for the *Evaluation* stance, accounting for the 93.30% of its expression. In comparison, adjectival collexemes were less frequently used for *Dynamic* and *Deontic* stance.

While adjectival collexemes are unambiguously evaluative, the meanings of the stance associated with the use of verbal collexemes are not always clear. Our research data agree with previous research findings that the verb groups of “emerge,” “appear,” “seem,” “occur,” and “strike,” reported in Francis et al. (1996, pp. 518–542; see also Herriman, 2000a), can, more or less, reflect the epistemic attitude of the writer-speakers. For example, verbs of “emerge” can indicate that “something is logically the case” (Francis et al., 1996, p. 519), though they can express Direct evidentiality as well. Likewise, verbal collexemes of the thinking group (e.g., *think* and *doubt*) denote a certainty or a likelihood judgment (Biber et al., 1999, p. 972; Biber, 2006).

Additionally, our research data suggest that reporting verbs are associated with evaluative meanings when used as collexemes of the INTRODUCTORY *IT* PATTERN. The use of *argue* serves as a good illustration. When used as a collexeme, *argue* can be pre-modified (42.86%) by the epistemic modal verbs *can* or *could*, or it can be used with no pre-modification by modal verbs (57.14%). The examples are shown in (3a) and (3b), respectively. To guarantee a better understanding of sample sentences, starting from (3a), (i) collexemes and other linguistic items crucial to stance expression are underlined, and (ii) the beginnings of the extraposed subject clauses are marked in **bold**. In the former case found in (3a), *argue* is pre-modified with *could* to express the epistemic possibility of the events/propositions introduced by the extraposed subject clauses. In the latter case [see (3b)], *argue* is used to deliver the Reported evidentiality.

(3) (a) It could, on the contrary, be argued **that** relations between the two kingdoms were relatively harmonious (w2a-010#027).
   (b) It has been argued **that** treating such people as ill, and admitting them to hospital for extended periods of time, is counter-productive and harmful in the long run…(w1a-007#093).

The detailed pre-modification information of reporting verbs is shown in Table 2. According to Table 2, most reporting verbs, including *argue*, *claim*, *admit*, *state*, and *say* [see (4a) and (4b)], can be pre-modified by the epistemic modal verbs (e.g., *can*, *could*, *may*, *might*) or obligation (semi-)modal verbs (e.g., *should*, *must*, *have to*) to express the epistemic possibility or deontic necessity of the events/propositions denoted in the extraposed subject clauses. Between the two types of judgments, the judgment of epistemic possibility is generally the more frequently observed with the use of reporting collexemes than that of deontic necessity (21.43 > 12.5%). In other cases, when these reporting verbs are not accompanied by modals or semi-modals [see (3b) or (4c)], they are not evaluative (Mair, 1990; Herriman, 2000a) but evidential.

**TABLE 2:** Modalized reported evidentiality.

Collexemes	Counts	CLS	Pre-modification (per cent)		No Modification
			by epistemic modals	by obligation modals	
*suggest*	13	15.82	0.00%	0.00%	100.00%
*agree*	10	10.83	0.00%	0.00%	100.00%
*argue*	7	9.42	42.86%	0.00%	57.14%
*claim*	5	5.71	40.00%	0.00%	60.00%
*admit*	4	4.96	0.00%	75.00%	25.00%
*state*	3	3.94	0.00%	33.33%	66.67%
*say*	14	3.93	50.00%	21.43%	28.57%
*Total*	*56*		*21.43%*	*12.50%*	*66.07%*

(4) (a) It could be said at any stage **that** more time and more efforts to find new developments upon the variety of peace plans… could have been used… (s2b-018#086).
   (b) It has to be said **that** some of the reason for this is that S&P rate only the top tier of companies in Europe (w2c-013#055).
   (c) It was said **that** there was a huge miserable urban proletariat with nowhere to go (s2b-025#081).

Although five of the seven reporting collexemes can be modalized, reporting collexemes generally express the less modalized than the non-modalized evidentiality (33.93 > 66.07%). Moreover, the reporting verbal collexemes that can be modalized possess lower CLSs than *suggest* and *agree*, which are the two reporting collexemes that are not modalized in our *corpus*. In other words, the prototypical reporting verbs attested in the INTRODUCTORY *IT* PATTERN are used to express a non-modalized Reported evidentiality. Therefore, although reporting verbal collexemes can be used for epistemic judgment, and, occasionally, for deontic judgment, they are primarily associated with the Reported evidentiality. Nonetheless, reporting verbal collexemes contribute to an enriched lexical diversity for the expression of various stance types.

Apart from the primary use for the expression of *Epistemic* stance, verbal collexemes can also be used to deliver various other stance types/subtypes. For instance, *hope* and *desire* are used for the expression of Volition, *take*, and *cost* for Circumstance, then *make sense*, *help*, and *matter* for various subtypes under the *Evaluation* stance.

Observations of the use of verbal collexemes have, thus, revealed their statistical and functional importance in decoding how the INTRODUCTORY *IT* PATTERN functions as a stance marker. The statistical importance lies in the fact that verbal collexemes are extensively deployed to express a *Epistemic* stance, the subtype of Circumstance and Volition, and are moderately deployed to express various subtypes of the *Evaluation* stance. The functional importance lies in their affordance to be used for evaluative purposes through their lexical meanings and the linguistic circumstance, especially the co-occurring modal verbs. Compared with research that solely focuses on adjectival collexemes of the INTRODUCTORY *IT* PATTERN (Van Linden, 2010; Hilpert, 2014), the additional analysis of the verbal collexemes offers a diversified and vital dimension for analyzing the stance meanings of the pattern. The following sections will then focus on reporting and discussing what verbal and adjectival collexemes can reveal about the stance meanings of the INTRODUCTORY *IT* PATTERN.

## Collexemes as the Semantic Basis for the Expression of Stance

**FIGURE 1:**
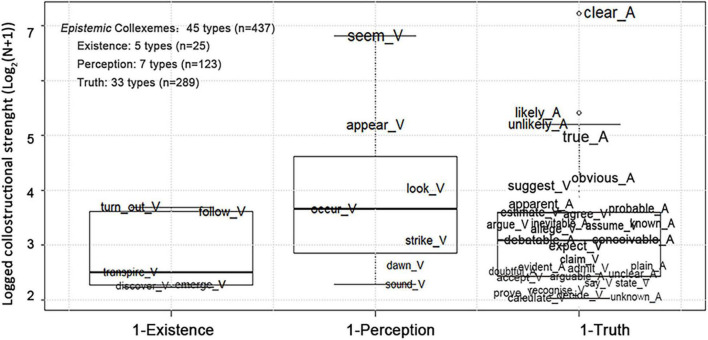
Logged collostructional strengths (CLSs) of the *Epistemic* collexemes.

**FIGURE 2:**
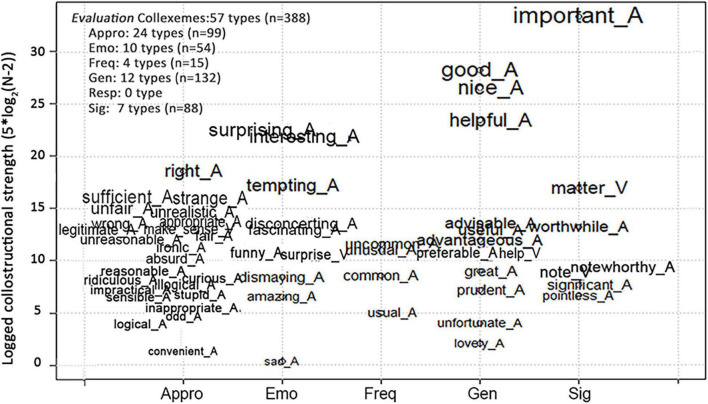
Logged CLSs of the *Evaluation* collexemes.

Detailed information about the uses of adjectival and verbal collexemes is reported and discussed in this section. Figures 1, 2 list the adjectival and verbal collexemes used to denote *Epistemic* and *Evaluation* stances, while those expressing *Deontic* and *Dynamic* stances are reported in Tables 3, 4. In Figures 1, 2, the logged CLS values of *Epistemic* and *Evaluation* collexemes are shown on the *y*-axes and are visualized by the font sizes of collexemes. Therefore, the collexemes with higher CLSs are placed higher and have larger font sizes. Moreover, for better readability, adjectival and verbal collexemes were marked with “_A” and “_V,” respectively. In addition, the space between phrasal verbs was replaced by “_.”

**TABLE 3:** Adjectival and verbal collexemes associated with *Deontic* stance.

Stance subtypes	Collexemes	Counts	CLS
Obligation	*necessary_A*	48	91.96
	*imperative_A*	4	10.22
	*essential_A*	5	7.36
Volition	*hope_V*	5	4.51
	*desire_V*	2	4.05

**TABLE 4:** The groupings of Ease-of-Performance collexemes.

Collexemes	Groups	CLS	Counts	Affirmation (per cent)	Negation (per cent)	Counts of difficulty
*possible*	potential	195.76	76	81.08%	**18.92%**	14
*impossible*	difficult	62.11	26	**100.00%**	0	26
*difficult*	difficult	137.74	73	**97.26%**	2.74%	71
*hard*	difficult	59.89	33	**100.00%**	0	33
*easy*	easy	66.73	37	91.89%	**8.11%**	3
*Total*			*245*			*147*

*Note: The percentages of difficulty assessment in the use of each Ease-of-Performance collexeme are marked in bold. For example, the 18.92% negated use of possible is also an expression assessing the difficulty of performance. Thus, this percentage is marked in bold.*

### The Collexemes Associated With *Epistemic* Stance

According to Table 1, *Epistemic* stance is the most frequently expressed stance type (*n* = 437), and Figure 1 reports the lexical basis for its linguistic realization. According to Figure 1, the collexemes most strongly associated with the expression of the Existence, Perception, and Truth subtypes are *turn out* (with the meaning of *appear to be*), the intransitive *seem*, and the adjectival *clear*, respectively.

To express the Existence subtype, collexemes (as reported in Figure 1) highlight a direct or indirect perception of the existence or coming into existence of events/propositions. The prototypical Existence collexeme *turn out*, possessing the highest CLSs among all Existence collexemes, primarily introduces the observed events/propositions. For example, in (5), the speaker was a judge analyzing a case of business’ secret infringement, where testimonies were needed to prove that the secret was indeed treated as such. In this case, the testimonies that would be directly perceived in the court could tell that *the plaintiff was right*. Hence, the perception verbal phrase *turn out* presents the observed propositions/events or expresses Direct evidentiality. The same function is also seen with *discover*. A few other Existence collexemes, *emerge* and *transpire*, can express both Direct and Indirect evidentiality. For example, the *emerge* in (6a) presents a proposition about the meaning of a word that is deducible from other evidence. Alternatively, in (6b), the *emerge* introduces visually observable events, thus, expressing a Direct evidentiality. The same multiple uses were observed with *transpire*.

Furthermore, another Existence collexeme, *follow*, is only used to express Indirect evidentiality. It displays Indirect evidentiality by indicating that propositions/events should happen rather than have happened or are observed. For example, the *follow* in sentence (7) is used to argue that an event *will* not necessarily happen because it is not supported with a convincing reasoning. In this case, the judgment is not based on a direct observation but on an indirect deduction. In other cases, *follow* is complemented by *from*-prepositional phrase (PP) to suggest the basis of reasoning, as in *it follows from that/the very terms of that/those assumptions*.

(5) It turns out, at the trial, **that** the plaintiff was right (s2a-066#060).
(6) (a) It emerged, however, **that** that the word *restraint* used by London and Washington did not necessarily mean taking no action (s2b-015#028).
   (b) It emerged later **that** he had bitten it (w2c-014#052).
(7) And when that happens, it doesn’t necessarily follow **that** slowly restoring the boundary conditions to their previous values will cause the climate to flip back into the state it used to be in (w2b-025#052).

According to the above results, the Existence subtype boils down to Direct and Indirect evidentiality as primarily actualized by verbs of perception. This finding is in line with Francis et al. (1996). Additionally, we found that the prototypical Existence collexeme, *turn out*, whose CLS is the highest, manifests Direct evidentiality while others feature Indirect, or both Indirect and Direct evidentiality. Hence, the expression of Existence *via* the INTRODUCTORY *IT* PATTERN features a mixed expression of evidentiality, which mainly features a non-modalized/Direct evidentiality.

Compared with Existence, the subtype of Perception put more emphasis on Indirect evidentiality, indicating that the perceived events/propositions originate from an indirectly observed evidence. Among Perception collexemes, the five collexemes with lower CLS values (see Figure 1) must be complemented to indicate indirect evidence or the lack of direct evidence. For example, *look* and *sound* are somehow complemented by the co-occurring subordinators, including *as if*, *as though*, and *like*, to present propositions/events that can only be felt or inferred to be the case/likely/true (Field, 1997; Dixon, 2005). Likewise, *occur* has to be complemented by *to*-PP, *strike* co-occurs with *me*, and *dawn* with *on*-phrase or *upon*-phrase to indicate that events/propositions are perceived in the inner world of writer-speakers. In comparison, the collexemes, *seem* and *appear*, can be interpreted as *seem to be true/the case/likely* or *appear to be the case*. Hence, they can deliver the probability/likelihood judgment without complementation. Nonetheless, *seem* can be complemented by *to*-PP (47.57%) to reveal the perceiver as a first-person perceiver [*me* for 96.55% *vs. us* for 3.45%; see (8)], while *appear* is almost invariantly used without any complementation and is associated with events/propositions expected to happen [see (9a) and (9b)].

The use of Perception collexemes and their complementation statistics entail different degrees of (inter)subjectivity in the expression of Indirect evidentiality or epistemic judgment. The expression can lean toward the intersubjective end by revealing the perceiver as a first-person or third-person perceiver (Whitt, 2011) *via* various forms of complementation, thus, highlighting a self-sourced or other-sourced perception. As the self-sourced perception is less reliable than the other-sourced perception, an expression of evidentiality that features the first-person perceiver, thus, entails higher subjectivity (Whitt, 2011). Most importantly, the Perception collexeme possessing the highest CLS value is *seem* (see Figure 1), which features a less solid observation of the inner world of reasoning/inference of the first-person perceiver. The high CLS of *seem* suggests that the INTRODUCTORY *IT* PATTERN favors more subjectivity in the expression of Perception.

(8) It seems to me **that** the Royal Family are, at best, marginal to tourism (s2b-032#043).
(9) (a) It would appear **that** Mr. Daniel’s case throughout was that there were some that had not been satisfied… (s2a-069#017).
   (b) It now appears **that** the area they were looking at will be occupied by various pieces of the equipment (w1b-021#055).

As opposed to Existence and Perception, the subtype of Truth displayed mixed modes of information presentation that either explicitly evaluates likelihood or exhibits a modalized Indirect evidentiality. The two modes are realized by adjectival and verbal Truth collexemes, respectively. Among adjectival Truth collexemes, *clear* has the highest CLS value, followed by *likely*/*unlikely*, and then by *obvious/true*. Among them, *clear* is frequently modified (25.64%) by intensifying degree modifiers, such as *quite* and *very* [see (10)], but is hardly modified (*n* = 1) by the totality reinforcing modifiers (e.g., *absolutely* or *definitely*). Moreover, it can be used in comparative form, as in *it could not be clearer*. Comparatively, *likely* and *unlikely* are more frequently pre-modified (31.82%) by a wider range of degree intensifiers, especially by comparative adverbials, *more* and *less*, to be used in a comparative form. According to Paradis (2001), modification by intensifying modifiers and by the comparative/superlative use of adjectives indicate their scalarized meanings. With reference to these standards by Paradis (2001), *clear* and *likely/unlikely* feature scalarized meaning. Moreover, *likely/unlike* manifests typical characteristics of scalarized meaning as they are more frequently used in the comparative form than *clear*. In contrast, adjectives with extreme meanings are modified by the totality reinforcing modifiers like *definitely* and *almost* (Paradis, 2001). Thus, *clear* possesses a scalarized meaning that leans toward the extreme end as it can be occasionally modified by the totality adverbial *perfectly.*

In comparison, *obvious* and *true* are the extreme Truth collexemes denoting a higher certainty because they are rarely modified by degree intensifiers and can be pre-modified by totality adverbials [see (10b)]. Thus, *obvious* and *true* are less typical of scalarized adjectives but more characteristic of extreme adjectives. Adding up to the scalar, there is also a “dubitative” likelihood judgment, as in *it is debatable* or *it is not clear*, functioning to discharge the epistemic obligation of writer-speakers (Marín-Arrese, 2021).

Compared to adjectival Truth collexemes, the verbal Truth collexemes indicate the source of evidence for events/propositions. Among these collexemes, verbal collexemes of reporting feature a moderately modalized Reported evidentiality (see the previous section for detail), while the remaining verbal Truth collexemes manifested a modalized Indirect evidentiality by suggesting the existence of some supporting evidence for the presented events/propositions. For *estimate* and *calculate*, the evidence is the certain measurement (Dixon, 2005). On the other hand, for *prove*, *accept* and *expect*, it is some convincing evidence, consensus, and generally known routines, respectively. Meanwhile, for *assume*, *allege*, and *recognize*, the reason is the certain personally held convictions over the likelihood of the presented events/propositions [see (11)]. Interestingly, these remaining verbal collexemes are rarely pre-modified by modal verbs to alter the expression of stance, like the case of reporting collexeme *say*. The occasional pre-modification simply functions to intensify or tone down the degree of certainty in the likelihood judgment, as in *it must be presumed that* or *it might be expected that*.

(10) (a) It is quite clear **that** the majority of the new religions attract their members from the privileged middle classes… (w2a-012#010).
     (b) It is perfectly true **that** this is a surprisingly tight package to many commentators (s1b-052#013).
(11) (a) It had been assumed **that** developments of this sort would please the government in Beijing (w2e-008#049).
     (b) It is assumed, if you work for the Royal Shakespeare Company **that** you have read the play or seen the play or even collaborated on a previous production (s1b-023#057).

Therefore, Existence, Perception, and Truth expressed by verbal collexemes, demonstrate evidentiality that is modalized to different degrees. Perception expresses fully modalized/Indirect evidentiality that features higher subjectivity, while Existence and Truth display a mixture of modalized and non-modalized evidentiality. The modalization is observed with verbal collexemes of existence and perception, reporting as well as verbs of cognitive activity despite the Direct/Reported evidentiality entailed in their lexical semantics. In some rare cases, reporting verbs are used to deliver deontic meanings (e.g., *it must be said/admitted that*), disconnected from the Indirect evidentiality or Reported evidentiality (Auwera and Plungian, 1998), resulting in a mere deontic assessment of events/propositions. A plausible conclusion is that non-evaluative *lexis* is associated with evaluative meanings when used as collexemes of the INTRODUCTORY *IT* PATTERN *via* the modalized evidentiality and the linguistic circumstance, especially one with modal verbs.

It is, therefore, inefficient and inaccurate to maintain the ternary division of *Epistemic* stance into Existence, Perception, and Truth because they fail to adequately describe the meanings or uses of the verbal collexemes. For example, verbs of existence/occurrence also express the inference/reasoning of writer-speakers, and, thus, should not simply deliver “Existence” but also the epistemic judgment of writer-speakers. Moreover, it should be noted that this epistemic judgment is within the domain of *Epistemic* stance while the existence/occurrence observation belongs to that of the evidentiality. Based on our research results, it will be more appropriate to have a bipartite division of the *Epistemic* stance into the Modalized Probability and General Probability associated with verbal collexemes and adjectival collexemes, respectively. The Modalized Probability is a likelihood judgment that also indicates the source of the judgment, including the inner perception [e.g., *seem*, *appear*, and *look/sound (as if)*], general reasoning/inference (e.g., *it can be argued*; and *it could be said*), assumption (e.g., *assume* and *allege*), certain measurement, and the routine. In comparison, General Probability is a general likelihood judgment featuring different degrees of certainty in the judgment. It favors an intermediate likelihood judgment typically expressed with *clear*, *likely*, and *unlikely*, which, in turn, reflects a lack of evidence and certainty (Haβler, 2010). The INTRODUCTORY *IT* PATTERN, thus, features a modalization cline of evidentiality ranging from (a) non-modalized observation or reporting, (b) grammatically marked modalization (e.g., *it can be said*), to (c) semantically oriented modalization actualized by verbal and adjectival predicates that point to a lack of direct access to events/propositions. Along this line, the expression of *Epistemic* stance by the INTRODUCTORY *IT* PATTERN is at the modalized end of the cline covering phases (b) and (c).

Besides, *Epistemic* stance demonstrates the tendency to display positive likelihood judgment. Details reported in Table 5 show that the *Epistemic* stance features an overall emphasis (86.04%) on a positive likelihood judgment. Moreover, such tendency is highlighted by verbal collexemes as they are almost solely used (above 93% occurrences) to express this positive judgment. In contrast, more adjectival *Epistemic* collexemes are used to express negative (12.89%; e.g., *it is hardly probable*) and neutral/dubitative (12.37%; e.g., *it is not clear*) likelihood judgment.

**TABLE 5:** The expression of *Epistemic* stance.

Collexemes	Positive		Negative		Neutral	
	Counts	%	Counts	%	Counts	%
Verbal (from Existence)	24	96.00%	0	0.00%	1	4.00%
Verbal (from Perception)	118	95.93%	5	4.07%	0	0.00%
Verbal (from Truth)	87	93.55%	6	6.45%	0	0.00%
Adjectival (from Truth)	145	74.74%	25	12.89%	24	12.37%
*Total*	*376*	*86.04%*	*36*	*8.24%*	*25*	*5.72%*

The prevalent positive likelihood judgment of the INTRODUCTORY *IT* PATTERN echoes the results of Hyland and Tse (2005), where a general positive *Epistemic* stance (55.5%) features the expression of *Epistemic* stance by the evaluative *that* construction. Interestingly, our findings show that the INTRODUCTORY *IT* PATTERN demonstrates a more robust tendency to express the positive *Epistemic* stance. Such tendency is more prevalently associated with Modalized Probability, indicating the overall tendency for modalized evidentiality to be affirmative.

### The Collexemes Associated With the *Deontic* Stance

Details reported in Table 3 show that the variety of collexemes expressing the *Deontic* stance was limited, with only five associated collexemes. Furthermore, observations of their use revealed that *Deontic* collexemes were associated with different modal verbs.

The expression of the Obligation subtype shows the strongest association with the adjectival collexeme *necessary*, which can be modified by possibility/permission/ability modal verbs, *may* [*n* = 5; see (12)] and *could* (*n* = 1), or prediction/volition modal verbs *will* (*n* = 2) and *would* (*n* = 1). However, no obligation modals or semi-modals, including *must*, *should*, and *have to*, are used with it. As for the other less attracted collexemes, such as *essential* and *imperative*, no pre-modifying modal verbs are seen. However, several main verbs of the extraposed subject clauses are pre-modified by the obligation modal, *must* [*n* = 1; see (13a)], and semi-modal, *have to* (*n* = 1). Besides, among the extraposed subject clauses that follow *imperative*, two cases of present subjunctive mood are found [see (13b); *n* = 2].

(12) It may be necessary **to** operate under a confidentiality agreement (w1b-030#031).
(13) (a) It is therefore essential **that** any room where gas is used must be adequately supplied with air, and must have an adequate flue to discharge the burnt gases (w2d-012#038).
    (b) It is, thus, imperative **that** the process of ratification be thoroughly completed (s1b-054#041).

For the two Volition collexemes, *hope* and *desire*, associations with different modal verbs are also found with their use. The verbal collexeme *hope* are followed by extraposed *that*-clauses (*n* = 4), and *that*-clause with *that* omitted (short as “zero *that*-clause”) (*n* = 1). All extraposed *that* clauses following *hope* have modal verbs that pre-modifies the main verb, including the volition/prediction modal *will*, and possibility/permission modals *might* and *can*. In contrast, the verbal collexeme *desire* uniformly occurs with the extraposed infinitival clauses [see (14)], where the use of modal verbs, subordinators, and grammatical subjects is blocked. Hence, the linguistic elements between *desire* and the main verb of the extraposed infinitival clause are reduced to a minimum. The eagerness connotated in *desire* is, thus, maintained.

(14) It may be desired **to** store all this information in a computerized database, but the cost of converting the data by hand can be prohibitive (w2a-032#044).

The above findings on the use of *Deontic* collexemes point to the different degrees of obligation and volition connotated in the Obligation and Volition collexemes. The concept of the degree of obligatoriness is mentioned in Nuyts (2005, p. 23) and in Van linden and Verstraete (2011). Likewise, the degrees of volition are also noted (Dixon, 2005). In this study, the varying degrees in expressing Obligation and Volition are evident from two formal clues: the pre-modifying modal verbs of the collexemes, and the main verbs in the extraposed subject clauses. The former provides circumstantial meanings that can be read into the connotations of the collexemes (Martin and White, 2005, p. 65), while the latter indicates the connotations of the collexemes as the modal meanings are harmonically assimilated into the collexemes/the main clause predicate heads (Lyons, 1977; Bybee et al., 1994, p. 214). Therefore, by referring to the two formal clues, the semantic connotations of collexemes can be deciphered. Thus, when used as collexemes of the INTRODUCTORY *IT* PATTERN, *necessary* possesses weaker obligatoriness than *essential* or *imperative* because only the latter are accompanied with modal verbs of obligations (*must*, *have to*), and *hope* entails weaker volition than *desire* as it is not modified by obligation modal verbs but co-occurs with modal verbs of possibility/permission (e.g., *can, could*, and *might*). The scalar difference in the expression of obligation and volition is in line with findings by Van linden and Verstraete (2011) and also by Dixon (2005).

In addition, our results show that collexemes with lower obligatoriness/volition (e.g., *necessary* and *hope*) have higher CLS values than those with higher obligatoriness/volition. According to Table 3, among Obligation collexemes, the CLS of *necessary* is 91.96 while those of *imperative* and *essential* are considerably lower: 10.22 and 7.36, respectively. Likewise, between the two Volition collexemes, the CLS of *hope*, which entails lower volition, is also higher than *desire*. Thus, the fair conclusion is that the INTRODUCTORY *IT* PATTERN favors collexemes with lower obligatoriness/volition for the expression of the *Deontic* stance. This lower obligatoriness/volition subsequently connotates less desirability on the part of the writer-speakers. The preference of the INTRODUCTORY *IT* PATTERN for lower obligation/volition aligns with its generally impersonal and objective nature (Collins, 1994; Gómez-González, 1997; Herriman, 2000a; Berman, 2004; Biber, 2006; Hamawand, 2007), resulting in a reserved or a negotiable expression of stance.

### The Collexemes Associated With the *Dynamic* Stance

The expression of the *Dynamic* stance builds on five collexemes that denote the subtype of Ease-of-Performance, and four collexemes that deliver the subtype of Circumstance. These *Dynamic* collexemes (ranked in descending order by their CLSs) are *possible, difficult, easy, hard*, and *impossible* for Ease-of-Performance, then *take*, *cost*, *safe*, and *dangerous* for Circumstance. Among the Ease-of-Performance collexemes, *possible* and *impossible* can express both Ease-of-Performance [see (15a)] and Truth [see (15b)]. Between them, 76 out of 100 occurrences of *possible* express Ease-of-Performance, and 26 out of 28 occurrences of *impossible* denote the same stance subtype. Thus, these two are primarily associated with Ease-of-Performance and are only discussed as Ease-of-Performance collexemes in this section.

(15) (a) It is possible **to** achieve it (s1b-054#011).
     (b) It is possible **that** those turtles, which escape the slick, will find themselves with nowhere to breed (w2b-029#101).

Observations on the use of Ease-of-Performance collexemes reveal how a limited set of collexemes can designate Ease-of-Performance. These collexemes form three natural meaning groups, presented in Table 4 that respectively assessed the evaluated events/propositions as easy, potential, and difficult/impossible. Reported together are the negation statistics, as negation also influences the groupings.

According to Table 4, the collexeme *possible*, categorized under the “potential” group, primarily (81.08%) assesses events/propositions as potentially realizable, but it can also be negated (18.92%) to denote difficulty/impossibility. Meanwhile, the three collexemes, *difficult*, *impossible*, and *hard*, are in the “difficult” group, and most of their occurrences (98.26, 100, and 100%, respectively) denoted the difficulty of performance. The “easy” group only contains *easy*. Although most of its uses as an Ease-of-Performance collexeme denoted the ease of performance, in a small fraction of its occurrences (8.11%), *easy* is negated to denote the difficulty of performance.

Therefore, although Ease-of-Performance collexemes are predominantly associated with one Ease-of-Performance assessment result (“easy,” “potential,” or “difficult”), they can all express the difficulty of performance. The total count of collexemes denoting the difficulty of performance, including the negated use of *possible* and *easy*, and the affirmative use of collexemes from the “difficult” group amounts to 147, constituting 60% occurrences of Ease-of-Performance collexemes. Hence, the prevalent meaning of the Ease-of-Performance subtype is difficulty-of-performance.

Meanwhile, for the subtype of Circumstance, no degrees or scalar changes in the stance expression are observed in the use of Circumstance collexemes. However, each Circumstance collexeme is associated with one major stance meaning. The two verbal collexemes, *take* and *cost*, are idiomatically used to depict the financial cost, labor, time, and other resources needed to realize the events/propositions denoted by the extraposed subject clauses. Moreover, 75.76% occurrences of *take* comment on the amount of time [see (16)], while *cost* is invariantly used to specify the financial costs [see (17)]. The two collexemes, *take* and *cost*, are, thus, supplementary for the expression of Circumstance. A much different aspect of Circumstance is shown by the two adjectival Circumstance collexemes, *safe* and *dangerous*, used to describe the general environments or the atmosphere related to realizing events/propositions.

(16) It took me an hour **to** arrange them in that lot (s1a-019#091).
(17) It costs me like a fiver more **to** come in for nine o’clock than it does if I come in for eleven (s1a-008#043).

Thus, the two subtypes of the *Dynamic* stance, Ease-of-Performance and Circumstance, emphasize different aspects of the empirical laws governing the realization of the events/proposition denoted by the extraposed subject clause. The subtype of Ease-of-Performance expresses the degrees of ease in actualizing events/propositions, leaning toward an overall difficulty-of-performance assessment. In contrast, no degrees of circumstantial requirements are observed in the expression of Circumstance, which is in line with the general non-scalar nature of circumstantial stance subtype (Nuyts, 2014). Instead, Circumstance collexemes, like *take* and *cost*, are conventionally used to assess the different resources required to actualize events/propositions, reflecting the highly idiomatic use of the INTRODUCTORY *IT* PATTERN (Mair, 1990, p. 23; Kaltenböck, 2005).

### The Collexemes Associated With the *Evaluation* Stance

The range of collexemes that express the *Evaluation* stance is reported in Figure 2. For better visualization of Figure 2, (i) the CLS values of collexemes were logged and amplified [5*log_2_(*N*-2)], and (ii) the names of stance subtypes were abbreviated to the initial three to five letters (e.g., “Appro” for “Appropriateness”).

According to Figure 2, *Evaluation* collexemes are rich in lexical variety, totaling 57 collexemes. Among all six subtypes of the *Evaluation* stance, Appropriateness features the highest diversity, constituting 42.11% of all the *Evaluation* collexemes. In comparison, other subtypes, especially the Frequency subtype, has considerably fewer lexical varieties (only four collexemes). Besides, the subtype of Responsibility is not expressed by any adjectival or verbal collexeme and, thus, is not shown in Figure 2.

Figure 2 also shows that some adjectival collexemes have extraordinarily high CLSs. They are *important*, *good*, *nice*, *helpful*, *surprising*, and *interesting*, with *important* scoring the highest. Due to space limitations, our discussion on *Evaluation* collexemes will be illustrated through those with the highest CLSs.

The most attracted *Evaluation* collexeme, *important*, is a Significance collexeme. While it invariantly comments events/propositions as significant, the significance assessment affords multiple behavior implications. When *important* is followed by infinitival clauses (65.63%), it obliges the reader-hearers to follow the instructions expressed in the infinitival clauses [see (a) and (b) in (18)]. In addition, contrary to the claim that *important* only obliges reader-hearers to actualize the content of infinitival clauses (Van linden and Verstraete, 2011), our results show that the deontic meaning of *important* is equally detectable in almost all cases (95.24%), where the extraposed subject clauses are *that*-clauses/zero *that*-clauses (32.81%). In (19a), for example, *important* is the deontic as it obliges the reader-hearers to actualize the events/propositions denoted by the extraposed subject clause. This deontic meaning is supported by the fact that *important* is compatible with obligation/necessity modal verbs, *should* and *must*, as in (19a″), but is unacceptable with the possibility/permission modals *can/could/might*, as in (19a″). Thus, *important* is primarily (96.88%) deontic and delivers deontic assessment toward the events/propositions expressed by both infinitival and *that*/zero *that* clauses. In the two cases where *important* do not deliver deontic meaning, it comments on events/propositions that do not need to be realized because they are past events [see (21(a)], or the answer to a *yes-no* question [see 21(b)]. In both cases, the *important* is evaluative like another Significance collexeme *significant* [see (21c)]. Nonetheless, the collexeme is primarily a *Deontic* collexeme.

(18) (a) It’s very important **to** remember that the evidence wasn’t heard in Orkney (s1b-030#077).
     (b) It is very important **to** look at development in its broadest sense (w1a-014#023).

(19) (a) It is important **that** you consult both your institution and LEA as soon as possible (w2d-003#062).
  (a′) It is important **that** you should/must consult both your institution and LEA as soon as possible.
  (a″) *It is important **that** you could/would/might consult both your institution and LEA as soon as possible.

(20) It should be noted also **that** the rate of protein turnover is influenced by the activity of the thyroid gland (w2a-024#028).

Similarly, the verbal Significance collexeme, *note*, invariantly used in the form of *should/must be noted* also delivers deontic meaning [see (20)] as it obliges reader-hearers to note the events/propositions encoded in the extraposed subject clauses. Hence, the two Significance collexemes, closely associated with the deontic meanings afforded by the obligation modal verb *should*, deliver *Deontic* stance.

(21) (a) It was important **that** we got a good result after losing badly on Saturday (w2c-014#019).
  (b) It is also very important of course as far as uh toxic substances as well, **whether** they can cross this epidermal layer or not (s2a-046#076).
  (c) It is significant **that** they thought it necessary to provide any justification at all (w2a-001#048).

As opposed to *important* and *note*, a sense of deontic meaning is only vaguely observable but equally deniable with the use of other Significance collexemes. For example, the *noteworthy* in (22b) comments events/proposition as worthy of notice rather than obliging reader-hearers to note. For other Significance collexemes, including verbal *matter* [see (22a)], *significant* [see (21c)], and *pointless* [see (22c)], the stance meaning is also more evaluative than deontic. Therefore, apart from *important*, Significance collexemes generally comment on the significance of events/propositions but do not oblige reader-hearers with regards to the assessed events/propositions.

(22) (a) It didn’t matter **what** it was (s1a-060#195).
  (b) It is noteworthy **that** his brother, William, swiftly paid off the original debt to James Karr, which had begun the process of imprisonment in the first place (w2b-006#021).
  (c) It seems pointless **to** have it without them (s1a-068#164).

Following the distinctive uses of *important* and *note* as opposed to other Significance collexemes, the natural conclusion is that *important* and *note* should be Obligation or *Deontic* collexemes, as opposed to a Significance collexeme, as proposed by Herriman (2000a) and Zhang (2015). In addition, *note* is associated with stronger obligatoriness than *important* as the former is constantly used for deontic meaning, while the latter can be used for evaluative purposes like other Significance collexemes [see 21(a) and 21(b)]. The relatively weaker deontic meaning connotated in *important* is also mentioned by Van linden and Verstraete (2011). Furthermore, our study shows that *important* possesses lower obligatoriness than *necessary*, with the latter observed to demonstrate a constant deontic use in our *corpus* just like *note*. Additionally, *important* has stronger attraction to the INTRODUCTORY *IT* PATTERN than *necessary* and *note* (101.21 > 91.96 > 5.44). Hence, with *important* and *note* categorized as Obligation collexemes, it still holds true that Obligation collexemes with weaker obligatoriness (*important* < *necessary* < *essential/imperative*/*note*) have stronger attractions to the INTRODUCTORY *IT* PATTERN.

The group of collexemes with the second highest CLSs are *good* [see (23a)], *nice*, and *helpful*, denoting General Evaluation. Among them, *good* has an observable deontic flavor (47.17%), when used in the comparative [see (23b)] or superlative form [see (23c)]. In comparison, *good* used in the base form is more evaluative than deontic [see (23a)], whereas, for most other General Evaluation collexemes, a hint of deontic meaning seems acceptable but equally deniable. For example, *nice* in sentences (24a) and (24c) is evaluative for commenting on events that already have happened or are happening. Alternatively, *nice* is deontic in (24b) and (24d), when the situational setting (a couple was discussing about refurbishing the room) can provoke the deontic meaning, as in (24b), or when the events/propositions are hypothetical or desired, as in (23d). Thus, the stance meanings of General Evaluation collexemes vary between favorability and deontic meaning.

(23) (a) It was good **to** hear you on the phone today (w1b-012#003).
  (b) It was better **to** put it in her name cos it would be cheaper (s1b-080#251).
  (c) It is best not **to** tangle with one of them (w2b-021#073).
(24) (a) It is nice for me **to** do something where I’m moving (s1a-002#006).
  (b) It’d be so nice **to** have a casement here because you could then put, well, at least one casement possibly two (s1b-073#004).
  (c) It’ll probably be rather nice actually **trying** to match that up (s1a-086#100).
  (d) It would be nice **if** you could come in January (w1b-014#064).

The collexemes with the third highest CLSs are Emotive Reaction collexemes, including *surprising* and *interesting*. The use of *surprising* together with the verbal collexeme, *surprise*, is primarily (71.43%) negated (e.g., *not surprising* and *hardly surprising*), assessing an event/proposition as expected, whereas *interesting* is used in the affirmative form to arouse the attention of reader-hearers (Hewings and Hewings, 2002) to the unexpected events/propositions. Thus, *surprising* and *interesting* are considered “expectedness” adjectives (Groom, 2005). However, expectedness is simply the most typically expressed Emotive Reaction. Other emotions also include *sad/dismaying/disconcerting* and *fascinating/amazing/tempting*.

The collexemes with the fourth highest CLSs are the three Appropriateness collexemes: adjectival *right*, *sufficient*, and *strange*. The use of *right* expresses polarized appropriateness judgment based on general conventions inferable from the context. For example, in (25a), *these actions* denote the outrageous misconduct by several police officers. In this case, the general conventions for appropriateness judgment are those of social justice. In (25b), the general conventions are principles or laws of economics/finance. As for *sufficient*, the convention is the general agreement on the appropriate amount or degree; for *strange*, it is the general conventions on normality.

(25) (a) The consequences would be such that every sensible person would say: “It cannot be right **that** these actions should go any further.” (w2e-007#091).
  (b) it is right… **not to** flood the home market… or any other international market with news of the horrors of war the graphic uh pictures and so on (s1b-031#102).
(26) (a) It was fairly common **to** use symbolic representations (s2a-060#091).
  (b) It is unusual **for** a device to gain widespread acceptance without a clear and verified model of its operation (w2a-034#007).

The group of collexemes whose CLSs are the lowest among all *Evaluation* collexemes is the Frequency collexemes. Frequency collexemes assess events/propositions as the standard or non-standard practice. For example, (26a) described a situation where symbolic representations were publicly acceptable, which is different from the latter period under discussion. In cases like (26b), a sense of inappropriateness is observable from the use of *unusual*, evaluating events/propositions as disobeying the commonly accepted standard.

*Evaluation* collexemes can be about various value judgments that result in deontic meanings, or they can simply deliver evaluative meanings. However, the boundary between these two meanings is vague. For example, the General Evaluation collexeme, *good*, when used in the forms of *better* or *best*, is deontic (Collins, 1994), but these forms can sometimes be evaluative (Herriman, 2000a). The same vague deontic reading can be perceived in the use of some Appropriateness collexemes, which is also observed by Van linden and Verstraete (2011).

The reasons behind the expression of vague deontic meanings have raised some interest in scholars. A possible explanation involves some “face work,” which modulates the expression of deontic meanings (Brown and Levinson, 1987; Nuyts et al., 2010), thus resulting in the blurry boundary between purely evaluative and deontic meanings. In this study, writer-speakers assess propositions/events as significant, favorable, acceptable, or standard practices as a polite alternative to suggest that members in the community of writer-speakers should follow the propositions, or rather actualize the assessed events to meet various general conventions. In this sense, the *Evaluation* stance demonstrates more politeness than the *Deontic* stance, as its deontic connotation is only vaguely perceivable. Hence, the overwhelming prevalence of the *Evaluation* stance is of pragmatical significance.

## Conclusion and Implication

This study investigates how stance is expressed through the INTRODUCTORY *IT* PATTERN. A data set of 1,514 adjectival and verbal expressions of stance were identified from the ICE-GB. Manual annotation of the stance of writer-speaker expressed in the main clause of the INTRODUCTORY *IT* PATTERN was performed based on a fine-grained analytical framework by Herriman (2000a). Moreover, lexemes occurring as the predicate heads in the main clause predicates of the INTRODUCTORY *IT* PATTERN are vital to the expression of stance. A collexeme analysis was then performed to identify the range of lexemes that are statistically correlated to the patterns (called “collexemes”). The discussions of these collexemes yield three major findings.

First, similar to adjectival collexemes, verbal collexemes of the INTRODUCTORY *IT* PATTERN, including verbs of various meaning groups and the non-evaluative reporting verbs, can be associated with evaluative meanings. Thus, the INTRODUCTORY *IT* PATTERN functions as an evaluative constructional context that modalized non-evaluative lexemes and attracts evaluative lexemes of different syntactic categories.

Second, a closer examination of adjectival and verbal collexemes reveals that they are complementary in stance expression. Adjectival collexemes are generally important and widely used for all types of stances, whereas verbal collexemes are also important for two major reasons. On the one hand, verbal collexemes are a primary lexical resource for the expression of the *Epistemic* stance and the subtypes of Volition and Circumstance. On the other hand, the lexical semantics of verbal collexemes, their linguistic circumstance, and the linguistic context are combined to indicate the means or the relational process related to the evaluated events/propositions. When expressing the *Epistemic* stance, verbal collexemes indicate the evidence (including some measurement, consensus, inference, or good reasons), obliging the writer-speakers to make the likelihood judgment, resulting in the expression of modalized evidentiality. The modalized evidentiality is different from a general likelihood judgment expressed by adjectival collexemes. Thus, the *Epistemic* stance forms two natural subtypes: Modalized Probability and General Probability, which can be suitable substitutes of the tripartite division of Perception, Existence, and Truth.

Third, the core meanings of each stance type and subtype loom clearly *via* the semantic analyses of collexemes. The *Epistemic* stance delivers a positive likelihood judgment from two perspectives: with and without an indication over the source of evidence for the epistemically evaluated events/propositions. Meanwhile, the *Deontic* stance favors the expression of weaker obligation and volition. The *Dynamic* stance evaluates two empirical conditions. One is the Ease-of-Performance that tends to evaluate events as difficult to fulfill, while the other is the empirical circumstance. The *Evaluation* stance covers a diversified range of value judgments, including personal affect, generalized favorability, acceptability, and significance. The judgments are generally evaluative but can be deontic in some cases.

The above findings point to the meanings of the INTRODUCTORY *IT* PATTERN. The first meaning is an active semantic indication of the stance-taking process. The pattern expresses considerably less non-modalized evidentiality that features the observation or quotation of events/proposition, than Modalized Probability, that features the perception/recognition of events/propositions within the inner world of reasoning/inference of the writer-speaker. Moreover, this Modalized Probability is, in turn, less frequently expressed than General Probability, which features a more subjective likelihood judgment unsupported with identifiable evidence. In other words, the INTRODUCTORY *IT* PATTERN is evaluative as it leans toward an overt expression of evaluative meanings over superficially non-evaluative meanings, and it attaches evaluative meanings to the non-evaluative *lexis* (Zhou and Chen, 2021).

The second meaning of the INTRODUCTORY *IT* PATTERN is a scalarized stance expression. Its expression of *Epistemic* stance features a tendency to demonstrate more intermediate General Probability and favors an intermediately subjective Modalized Probability. Meanwhile, the pattern favors collexemes entailing weak volition/obligation, such as *important* and *necessary*, as opposed to collexemes of higher obligatoriness, such as *essential* and *imperative*. The same scalarized stance expression is also found with the subtype of Ease-of-Performance under *Dynamic* stance, leaning toward a difficulty-of-performance assessment. This scalarized expression of stance is considered generally existent in the expression of *Epistemic* and *Deontic* stance (Field, 1997; Biber et al., 1999; Nuyts, 2005, 2014; Whitt, 2011; Pérez Blanco, 2020), and is also observable with the adjectival predicates of the INTRODUCTORY *IT* PATTERN (Hilpert, 2014). However, our research findings show that the INTRODUCTORY *IT* PATTERN is especially reflective of the scalarized nature of stance types, including *Epistemic* and *Deontic* stance, and extending to the subtype of Ease-of-Performance. Moreover, the INTRODUCTORY *IT* PATTERN favors particular scalars in the expression of stance types/subtypes, such as an intermediate General Probability, difficulty-of-performance for the Ease-of-Performance, or a generally weak expression of *Deontic* stance.

The third meaning of the INTRODUCTORY *IT* PATTERN is a positive likelihood judgment, far more intensive than other stance devices like the evaluative *that* construction (Hyland and Tse, 2005). This positive likelihood judgment exemplifies the Pollyanna hypothesis (Boucher and Osgood, 1969), or the universal positive bias (Dodds et al., 2015), where linguistic expressions favor positive over negative expressions.

Overall, this study presents a picture of how lexical and grammatical features are interwoven to express the writer-speaker stance. It contributes toward decoding the stance marking functions of the INTRODUCTORY *IT* PATTERN at the lexical and grammatical levels. This study is also a methodological attempt to add a thorough semantic analysis of collexemes to a quantitative *corpus* research. Results are of referential value to the contextualized use of the INTRODUCTORY *IT* PATTERN (e.g., Charles, 2006; Zhang, 2015; Dong and Jiang, 2019) and could guide English learners toward its appropriate uses for stance expression. However, the limited sample size means that the diversity of all possible uses of the pattern might not have been thoroughly represented. Hence, the findings and conclusions should be interpreted with caution and with reference to observations of other researchers. Further research can explore the meanings of the INTRODUCTORY *IT* PATTERN by comparing its stance meanings in different registers and in text genres, or by adopting different research methods, such as sentence completion tests, to verify how language users conceptualize its meanings.

## Data Availability Statement

The datasets presented in this study can be found in online repositories. The names of the repository/repositories and accession number(s) can be found below: https://osf.io/6ndks/?view_only=c1f4f46376544b109a5501220e311cca.

## Ethics Statement

The studies involving human participants were reviewed and approved by Ethics Committee of the Department of Linguistics and Translation, City University of Hong Kong. Written informed consent for participation was not required for this study in accordance with the national legislation and the institutional requirements.

## Author Contributions

ZW proposed the main ideas, formulated the research questions, designed the data collection, performed the data analysis, and drafted the manuscript. WF and AF supervised the whole research process and helped narrow down the research goal. WF helped with reorganizing the first draft. AF critically reviewed the technical/operational premises of this research and carefully checked its theoretical foundations. All authors contributed to the article and approved the submitted version.

## Conflict of Interest

The authors declare that the research was conducted in the absence of any commercial or financial relationships that could be construed as a potential conflict of interest.

## Publisher’s Note

All claims expressed in this article are solely those of the authors and do not necessarily represent those of their affiliated organizations, or those of the publisher, the editors and the reviewers. Any product that may be evaluated in this article, or claim that may be made by its manufacturer, is not guaranteed or endorsed by the publisher.
